# Remaining forests on the Central Highlands of Madagascar—Endemic and endangered aquatic beetle fauna uncovered

**DOI:** 10.1002/ece3.9580

**Published:** 2022-12-12

**Authors:** Tolotra Ranarilalatiana, Herisolo Andrianiaina Razafindraleva, Gustaf Granath, Rasa Bukontaite Malm, Jean Claude Rakotonirina, Victor Razafindranaivo, Lala Harivelo Raveloson Ravaomanarivo, Frank Johansson, Johannes Bergsten

**Affiliations:** ^1^ Department of Entomology, Faculty of Sciences Antananarivo University Antananarivo Madagascar; ^2^ Department of Ecology and Genetics Uppsala University Uppsala Sweden; ^3^ Department of Bioinformatics and Genetics Swedish Museum of Natural History Stockholm Sweden; ^4^ Department of Zoology Swedish Museum of Natural History Stockholm Sweden

**Keywords:** biodiversity, conservation, deforestation, endemism, freshwater, species richness

## Abstract

Madagascar is known for its high endemism and as many as 90% of this unique diversity are forest‐dwellers. Unfortunately, the forest cover of Madagascar is decreasing at an alarming rate. This decrease can also affect aquatic insects, but our knowledge on aquatic insect diversity and distribution on Madagascar are limited. Although the eastern rainforests are considered the most diverse, the Central Highlands of Madagascar also harbors unique microendemic fauna but has been less studied. Here, we analyze the aquatic Adephaga beetle fauna of three remaining protected forests of the Central Highlands. Diversity, abundance, and uniqueness are compared between and within natural forests and surrounding grasslands. At least 15 undescribed species were found, highlighting the Central Highlands as an important area for endemism. The natural forests and the surrounding grasslands differed significantly in species assemblages. Interestingly, the three remaining forests differed in their assemblages with the geographically more distant Manjakatompo Ankaratra having the most unique fauna but also the highest altitude span. By contrast, the species composition was similar between the peripheral zones of each of the three remaining forests. The similarity of the fauna in the peripheral open habitats illustrates how some local forest endemics are replaced with widespread generalists in degraded habitats. Our study shows that the remaining forests of the Central Highlands of Madagascar are important refuges of unique fauna at high risk of extinction.

## Graphical abstract

We show that the aquatic beetle fauna in three remaining forest fragments on the Central Highlands of Madagascar shows local uniqueness with a high degree of undescribed species. These vestiges of a once more widespread highland forest are the last resorts for a unique highland fauna of high conservation priority. The fauna in adjacent grassland savannah is more similar and generalized, which means locally unique species disappear following deforestation.

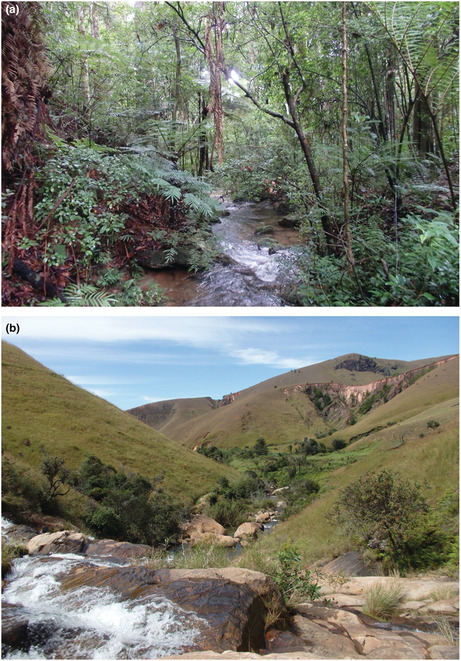

## 1 INTRODUCTION

Madagascar harbors exceptional levels of endemism and is classified as a biodiversity hotspot (Myers et al., 2000). The long geological history of isolation in combination with a great variety of topography, geology, and climate are the main factors behind this richness (Brown et al., 2014; Ganzhorn et al., 2014; Vences et al., 2009). As much as 90% of Madagascar's endemic diversity is associated with forests (Allnutt et al., 2008; Dufils, 2003; Goodman & Benstead, 2005). The majority of these species are found in native undisturbed forests, although a subset can survive in disturbed forests, secondary vegetation, and grasslands (see Irwin et al., 2010 for a review). The high level of deforestation in Madagascar, historically and currently, is therefore of greatest concern for conservation (Allnutt et al., 2008; Green & Sussman, 1990; Vieilledent et al., 2018a). A recent study found that Madagascar has lost 44% of its forest cover between 1953 and 2014 and that the deforestation rate was increasing alarmingly in the later years measured (99,000 ha/year in 2011–2014; Vieilledent et al., 2018a). For the period 2015–2017, this trend had accelerated further, reaching levels of 162,000 ha/year with roughly half of Madagascar's remaining and fragmented forests now being less than 100 m from a forest edge (Vieilledent et al., 2018b). With the average deforestation rate in the period 2010–2019, undisturbed humid forests in Madagascar are predicted to have completely disappeared in less than 30 years (Vancutsem et al., 2021).

**FIGURE 1:**
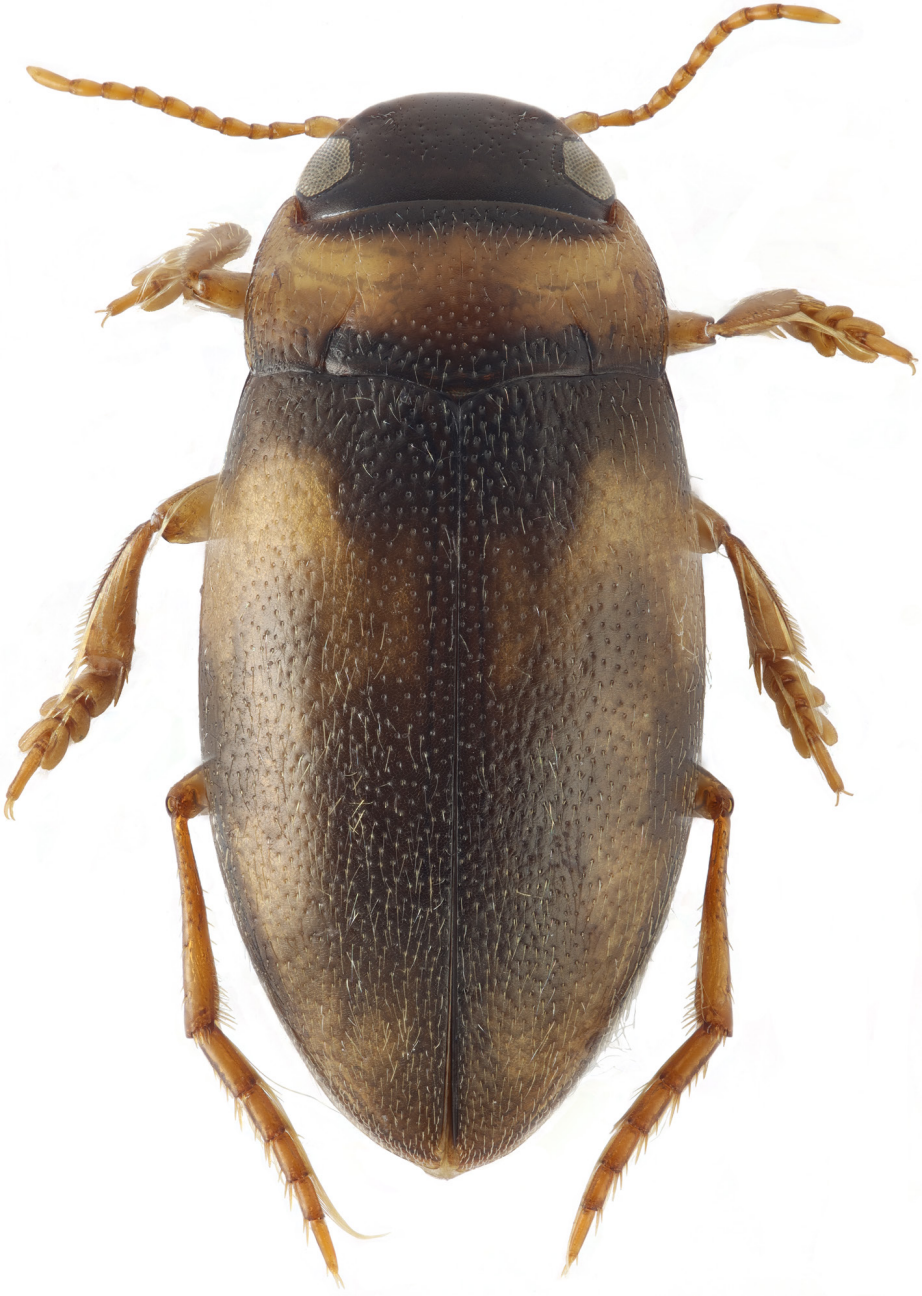
One of the many undescribed species and local endemics of the Central Highlands of Madagascar. This is a new diving beetle species of the genus *Uvarus* (Dytiscidae) from Manjakatompo Ankaratra. Length 2.6 mm.

Beta‐diversity, or species turnover, is generally very high in Madagascar, as most endemics are restricted to specific geographical regions on the island. Conservationist's concern with forest fragmentation is therefore not only a matter of fragment size, numbers, and interpatch distance but also of geographical location. The eastern rainforest biome is generally regarded as the most diverse and many of Madagascar's protected areas are distributed along the eastern escarpments. One interesting geographic area that has received little attention, is the Central Highlands of Madagascar. Even if the remaining forests here are very small, these remnants harbor significant highland‐endemic diversity (Figure 1; Andreone et al., 2007; Hjalmarsson et al., 2013; Raxworthy & Nussbaum, 1996).

The Central Highlands is today largely open secondary grasslands with few remaining forest fragments. The largest remaining forests are Manjakatompo Ankaratra, Ambohitantely, and Anjozorobe‐Angavo (Goodman et al., 1996). It is debated whether all of the Central Highlands was entirely forested before humans arrived, or if open grasslands may have existed prior to human colonization (Bond et al., 2008; Godfrey & Crowley, 2016; McConnell & Kull, 2014; Vorontsova et al., 2016). High endemism levels, lineage origination since Miocene, and regionalization of grasses (Bond et al., 2008; Hackel et al., 2018; Vorontsova et al., 2016) as well as evidence of pre‐human retraction of forests (Quéméré et al., 2012), suggest that some areas were naturally at least partly open land before humans arrived. The Central Highlands might thus have been a mosaic of forests, woodland, and more open savannah. However, the great expansion of grasslands at the expense of forest cover is indisputable in recent historical times (Crowley & Samonds, 2013; Godfrey et al., 2019). Multiple lines of evidence show a rapid biotic change around one to one and a half millennia ago (Burns et al., 2016; human presence on the islands is confirmed for a minimum of 2000 years: Douglass et al., 2019). Charcoal becomes much more prominent in layers from this time (Burney et al., 2003), C4 grasses expanded at the expense of C3 woody plants (Burns et al., 2016), and the megafauna collapsed (Godfrey et al., 2019; Goodman & Jungers, 2014).

The dire effect of deforestation and forest fragmentation on terrestrial endemic fauna in Madagascar, especially mammals (Craul et al., 2009; Crowley et al., 2018; Dunham et al., 2008; Goodman & Rakotondravony, 2000), birds (Andrianarimisa et al., 2000; Langrand, 1995; Langrand & Wilmé, 1997), reptiles (Jenkins et al., 2014; Lehtinen et al., 2003; Lehtinen & Ramanamanjato, 2006) and amphibians (Vallan, 2000), is well‐documented (see review by Irwin et al., 2010). The effect on freshwater ecosystems, however, and especially the invertebrate fauna, is rudimentarily known (Bamford et al., 2017; Benstead, De Rham, et al., 2003; Irwin et al., 2010). In Madagascar, the aquatic fauna shows equally high endemism levels as the terrestrial fauna (Elouard & Gibon, 2003; Vuataz et al., 2013), but knowledge lags behind, and conservation focus has remained on terrestrial systems (Bamford et al., 2017). A recent assessment of five freshwater groups (fishes, mollusks decapods, odonates, and plants) found that 43% of the 653 assessed species were threatened with extinction (IUCN categories CR, EN or VU, Méiz‐Tomé et al., 2018). For other groups of aquatic insects, the taxonomic knowledge in Madagascar is in itself very restricted, hampering any study or risk assessment.

There were two main aims with our study. The first was to compare the aquatic Adephaga beetle fauna of three forest vestiges of the Central Highlands. Such a comparison will provide information on how these areas overlap in species assemblages and which area that has the most unique invertebrate fauna. The second aim was to compare the species assemblages of the forest fragments with the surrounding savannah/grassland in the nonprotected zone outside each protected forest area. This information allows us to compare the difference in species assemblages between forests vs savannah/grassland and to examine to what degree forest‐dwelling endemic species survive in deforested habitats.

## 2 MATERIALS AND METHODS

### 2.1 Study area

**FIGURE 2:**
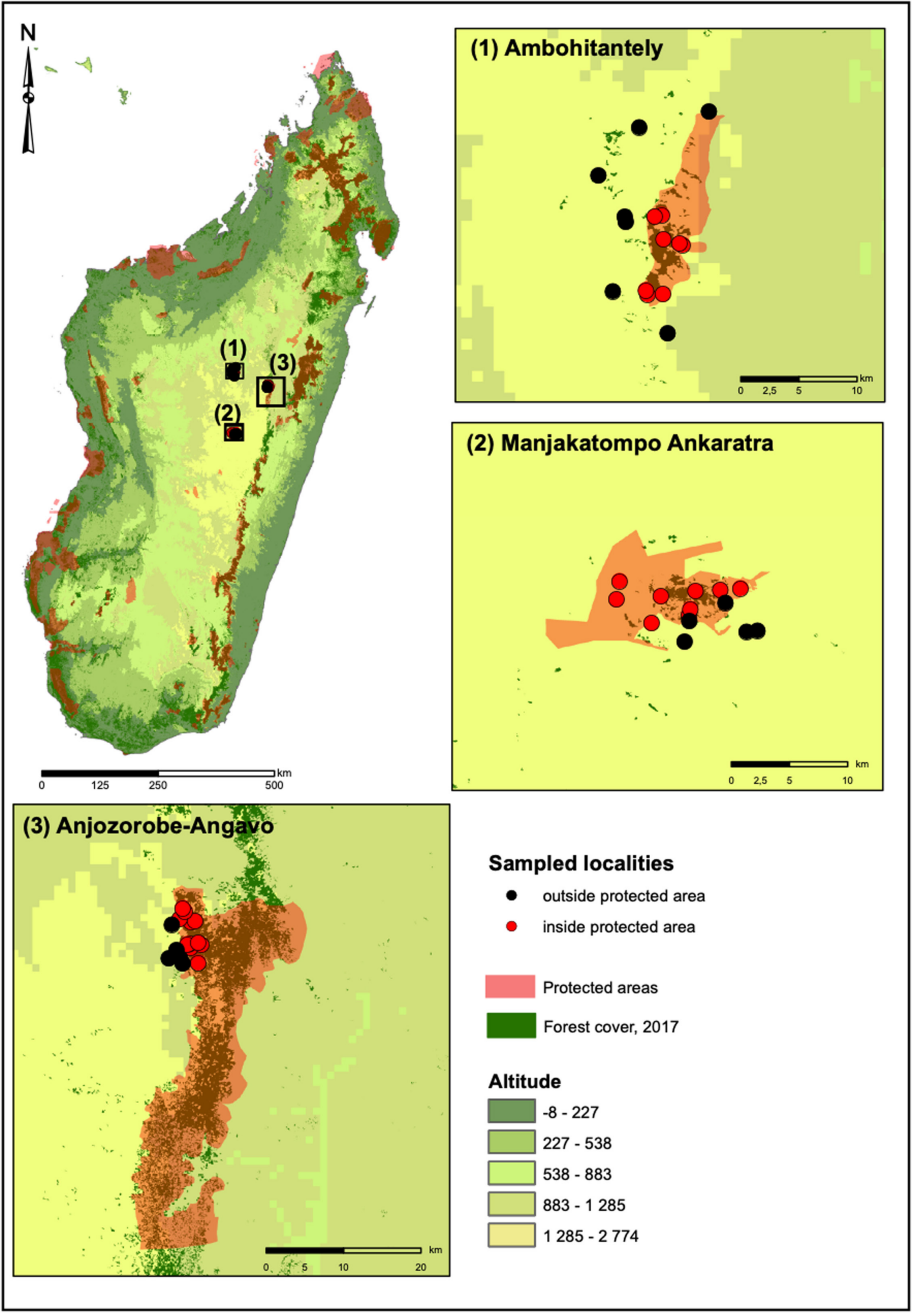
Map of the study areas on the Central Highlands of Madagascar. Protected areas follow Goodman et al. (2018) and remaining forest cover follows Vieilledent et al. (2018b) updated supplementary file over forest cover for 2017.

The Central Highlands covers a large part of the island and takes on the west of the North–South running eastern escarpment and declines gently towards the west (Andrianarimisa et al., 2000; Goodman et al., 1996). The Highlands region represent over 40% of Madagascar with an elevation above 800 m. The study was conducted in the three separate remaining forests Manjakatompo Ankaratra, Ambohitantely, and Anjozorobe‐Angavo, all situated in the central portions of the Highlands (Figure 2). Brief descriptions of these areas are provided below, while Goodman et al. (2018) provide more detailed descriptions.

#### 2.1.1 Manjakatompo Ankaratra

The forest part of Manjakatompo Ankaratra (official name: Réserve de Ressources Naturelles de Manjakatompo Ankaratra) is located on the eastern slopes of the Ankaratra Massif. The reserve covers an area of 8130 ha and has a very high range in altitude starting from about 1500 m to 2643 m. The forest has been under severe anthropogenic pressures lately, heavy deforestation for charcoal production and regular fires (Goodman et al., 2018; Hjalmarsson et al., 2013; Rabemananjara et al., 2012; Ranarilalatiana et al., 2019). The moist and evergreen forested parts are mostly between 1600 and 2000 m, and above 2000 m extensive grasslands extend with some pockets of ericoid thicket and bushes, heathland, and montane wetlands (Goodman et al., 2018; Guillaumet et al., 2008; Vences et al., 2002). The climate is moist and cool with cold and dry austral winter, and warm and wet austral summer (Vences et al., 2002). The average annual rainfall is 1424 mm (1981–2017), which falls mainly between November and April (Goodman et al., 2018).

#### 2.1.2 Ambohitantely

Ambohitantely (official name: Réserve Spéciale d'Ambohitantely) is located on the Central Highland of Madagascar on a distinct geological formation known as the Tampoketsa d'Ankazobe plateau. It has been legally protected since 1951. The reserve covers 4950 ha (5600 ha based on the decree in force) and consists of 80 forest fragments (Goodman et al., 2018), the largest currently 1160 ha (Goodman et al., 2018; Langrand, 1995, 2003). Apart from the natural forest fragments, some 35% of the reserve area is made up of grasslands and approximately 15% of exotic tree plantations, mainly *Eucalyptus* and *Pinus* (Bastian, 1964; Langrand, 1995). The forest fragments of Ambohitantely are among the last natural forest habitats in the central portions of the Central Highlands (Goodman et al., 2018). The largest forest fragment lies between 1550 and 1660 m in altitude (Langrand, 1995, 2003), while more than half of the reserve area is in the 1251–1500 m altitudinal zone (Goodman et al., 2018). The forests of Ambohitantely are classified as medium altitude moist evergreen forest with flora showing clear affinities to the eastern escarpment (Goodman et al., 2018). The climate is cool and sub‐humid with an average rainfall of 1461 mm (1981–2017, Goodman et al., 2018) that mainly falls between November and April.

#### 2.1.3 Anjozorobe‐Angavo

The Anjozorobe‐Angavo forest corridor (official name: Paysage Harmonieux Protégé du Complexe Anjozorobe‐Angavo) stretches north–south along the rim between eastern and western drainages about 45 (S end) to 70 (N end) km northeast of Antananarivo. As a larger (until recently) relatively intact forest, it has been used as a control or reference site to fragments of Ambohitantely in several vertebrate studies on the effects of forest fragmentation and habitat patch size (Goodman & Rakotondravony, 2000; Langrand & Wilmé, 1997; Vallan, 2000). The protected area covers 41,100 ha with more than half of the area in the altitude zone between 1251 and 1500 m (Goodman et al., 2018). The natural forest vegetation, currently comprising 28,000 ha (Vololonirainy & Mietton, 2013), is classified as a medium altitude moist evergreen forest (Goodman et al., 2018) and represents an important and unique transition between the eastern escarpment humid forests and the remaining forests on the Central Highland. From 1996 to 2016 Anjozorobe‐Angavo lost 33.2% of the forest cover, mainly in the southern portions of what would become the protected area (Goodman et al., 2018). Anjozorobe‐Angavo has an average annual rainfall of 1558 mm (1981–2017) most of which falls between November and April.

### 2.2 Fieldwork and sampling

We sampled water beetles of four families: Dytiscidae (diving beetles), Gyrinidae (whirligig beetles), Noteridae (burrowing water beetles), and Haliplidae (crawling water beetles). All belong to the Adephaga suborder of beetles. The habitats were classified as lotic (running water), lentic (standing water) or hygropetric (a thin water film on wet rocks by, e.g., seepages and cascades).

**FIGURE 3:**
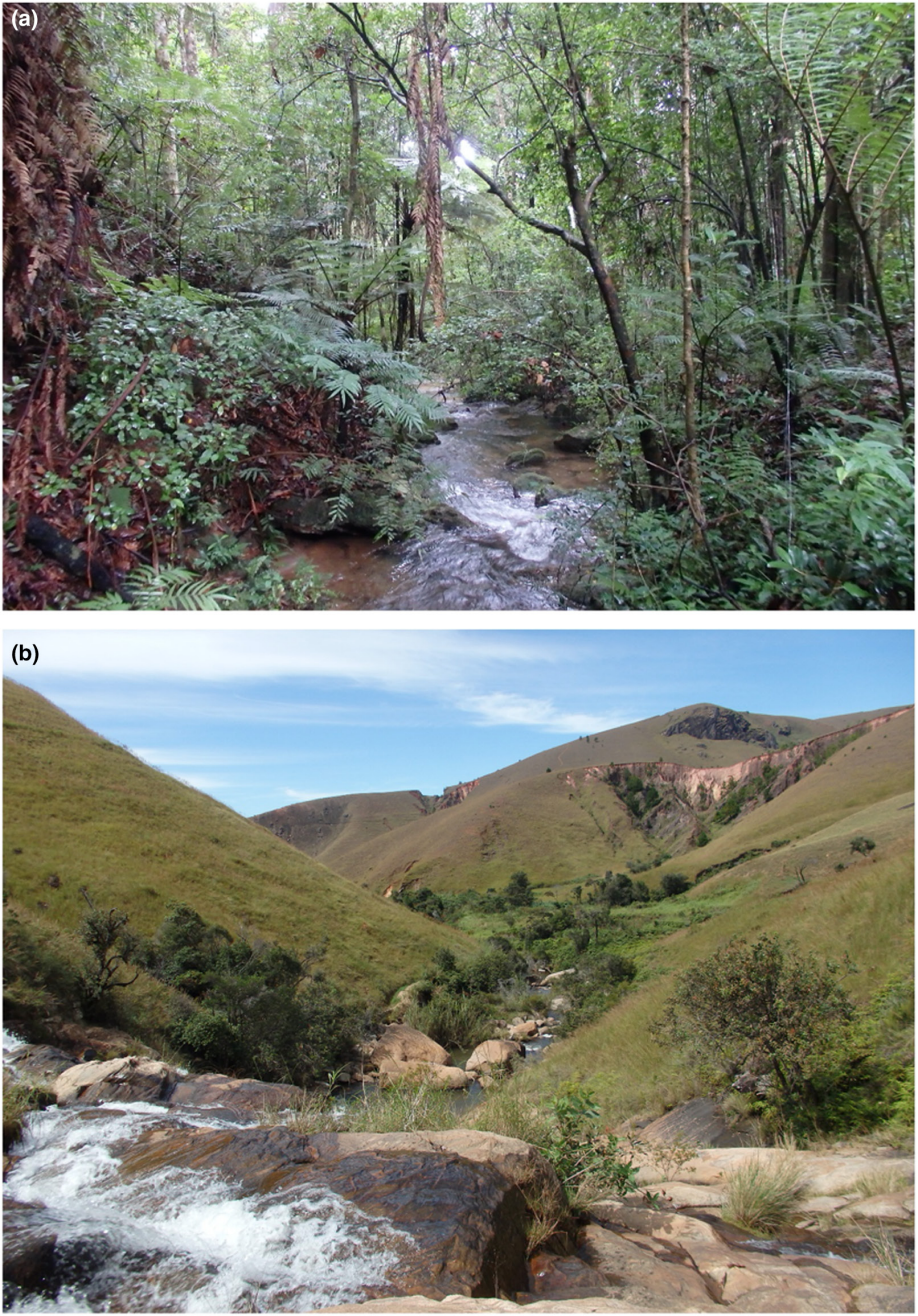
Two of the sampling localities inside and outside Ambohitantely: (a) forest stream inside reserve (MAD16‐21), (b) savannah stream in the peripheral zone outside the reserve (MAD16‐25).

Field work was done during the rainy season in February–April 2016, and localities inside and outside the protected areas were sampled for each of the three forest blocks (Figure 3, Tables A3, A4, A5). Localities inside protected area boundaries largely correspond to forested habitats and outside protected areas to deforested habitats except for Manjakatompo Ankaratra where several localities within the protected area emphasized its advanced state of degradation, and in the alpine zone of the protected area above 2000 m, there is no forest cover. For the larger Anjozorobe‐Angavo, the sampling was restricted to the northwestern part of the protected area. We sampled a minimum of five localities inside and five outside the protected area boundaries and focus on lotic environments, but a few localities were lentic and one hygropetric (the latter excluded from statistical analyses, see below).

The collecting method consisted of a semi‐quantitative sampling approach. For each locality, the sampling time was around 2 h, and all aquatic Adephaga specimens recovered in the net were sampled to give an abundance measure per species. The specific sampling positions at each locality were chosen to cover the different types of microhabitats. Sampling was done using a hand water net (0.5 mm mesh size) and sieves with different sizes, depending on the water body size and the type of microhabitat. Specimens were preserved in labeled plastic tubes containing 95% ethanol. The samples were stored in a laboratory refrigerator for subsequent sorting and identification.

### 2.3 Sample preparation and identification

All samples were sorted and identified to species level using identification keys (listed in Bergsten et al., 2022 per genus) and original descriptions from available literature and reference collections. If needed genitalia were extracted with fine forceps or a pin from the tip of the abdomen and glued onto a card together with the dry‐mounted specimen for examination under a microscope (Leica MZ12.5). Specimens are stored at the Swedish Museum of Natural History, Stockholm. The samples included several undescribed species, and these have been numbered as morphospecies 1, 2, 3, sometimes with a “working name” for reference within quotation marks. None of the names herein used are issued for the public and permanent scientific records or for purposes of zoological nomenclature (ICZN article 8.2). This also applies to the “working names” of undescribed species that will be formally described elsewhere. A few species belong to species complexes yet to be resolved and here the informal term cf. is used. It is here to be understood as indicating affinity to the named species following cf. but may or may not be conspecific.

### 2.4 Statistical analysis

Differences in species richness among areas (the three reserves) and location (inside the protected forest and outside in the peripheral zones) were investigated using rarefaction curves with the iNEXT package (Chao et al., 2014; Hsieh et al., 2016, 2022) in R statistical software (ver 4.2.1 R Core Team, 2022). Curves were fitted using sampling‐units incidence matrices that indicate the presence/absence of species. These curves were extrapolated to the total number of samples in an area for inside/outside curves, and to 20 localities for area curves to better show the general trajectories. We used 50 bootstrap replications (the default) to create confidence intervals around the curves.

To compare species assemblages between the forest reserves and between forest reserves and peripheral zones we performed multivariate (multi‐species) analyses. First, we fitted generalized linear models (GLMs) with a negative binomial distribution using the package mvbund (Wang et al., 2012, 2022). Community differences among areas, location, and their interaction were tested with a log‐likelihood ratio test assuming the independence of species response variables (Wang et al., 2012). We performed “species‐by‐species” univariate tests to further explore individual species responses. To complement the GLM approach, we also performed a distance‐based test, namely a PERMANOVA based on Bray‐Curtis dissimilarities (distance matrix) using the package vegan (Oksanen et al., 2019). The same dissimilarity distance matrix was used to test for differences in beta‐diversity (Anderson et al., 2006). To visualize the separation of species community across areas and locations, we used a nonmetric multidimensional scaling (NMDS), with a Wisconsin double standardization that is a gradient analysis based on a distance matrix.

## 3 RESULTS

**FIGURE 4:**
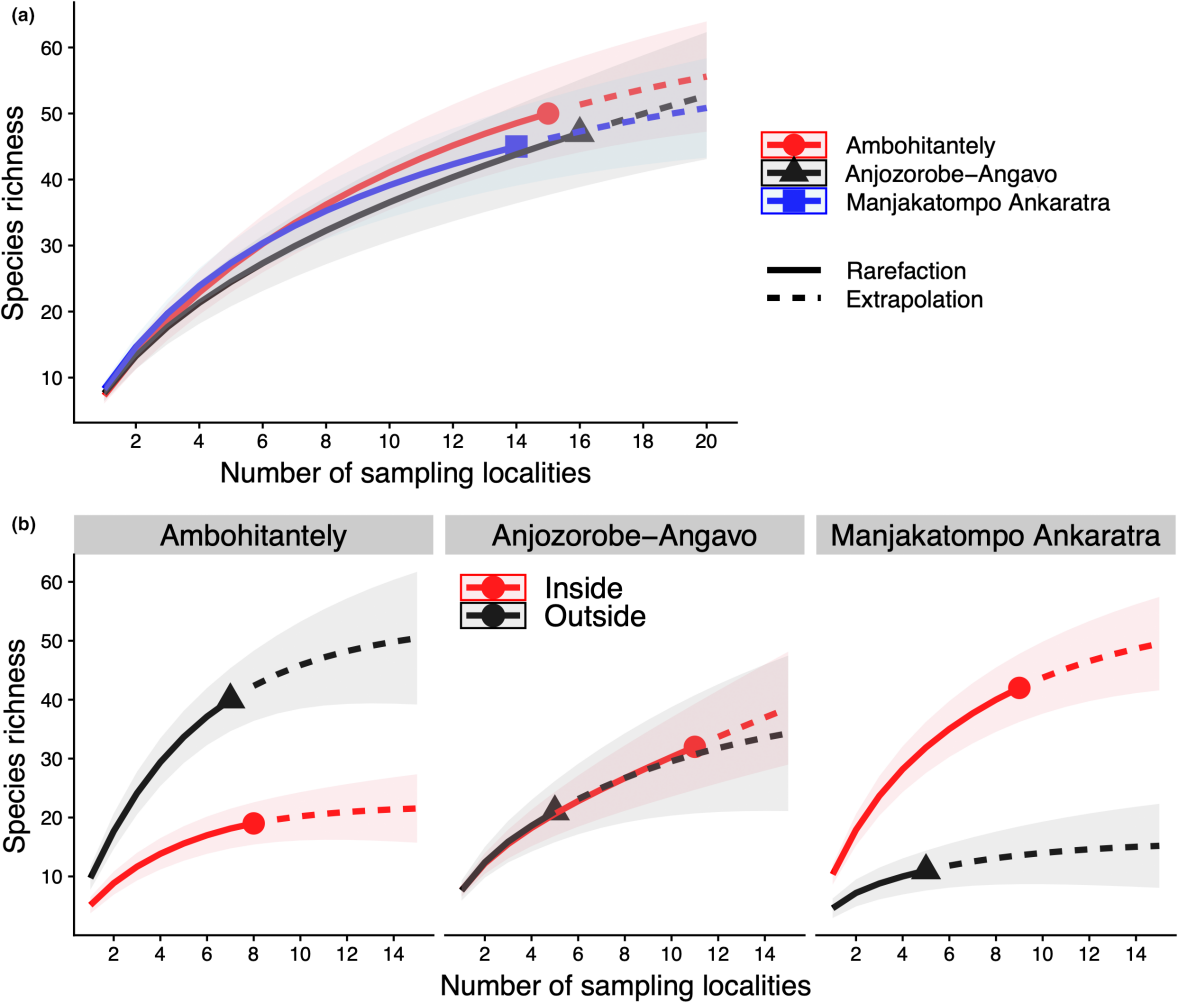
Species richness rarefaction curves for (a) each area (Ambohitantely, Anjozorobe‐Angavo, Manjakatompo Ankaratra) and (b) inside or outside the reserve at each area. Shaded areas represent a 95% confidence interval.

3463 individuals of aquatic Adephaga were sampled across 44 localities representing 92 species, of which 74 belong to the family Dytiscidae, 12 to Gyrinidae, 5 to Noteridae, and 1 to Haliplidae (Table 1). In total 28 species were recorded at only one locality. The total number of species, as well as rarefied species richness, was very similar between the three areas (46, 47, and 48 species; Figure 4a, Tables A6, A7, A8). For Manjakatompo Ankaratra, the sampled species richness and rarefied species richness were higher inside than outside the protected area boundaries (42:12; Figure 4b, Table A6), but for Ambohitantely, the pattern was the opposite (18:39; Figure 4b, Table A7). For Anjozorobe‐Angavo, species richness was higher inside than outside the protected area (32:21; Table A8) but rarefied species richness was equal (Figure 4b). Rarefaction based on individuals showed similar results (results not shown). Overall, 41 species (45% of all species) were only found inside the reserves, and 18 (20% of all species) were only found outside the reserves.

**TABLE 1:** Species and number of individuals of aquatic Adephaga (Dytiscidae, Gyrinidae, Haliplidae, Noteridae) collected in the three investigated areas.

Taxon	Endemic to Madagascar	Manjakatompo Ankaratra	Ambohitantely	Anjozorobe‐Angavo	Inds.
Dytiscidae					
Colymbetinae					
*Rhantus bouvieri* Régimbart, 1900	Yes	90		1	91
*Rhantus latus* (Fairmaire, 1869)	Yes	15	1	1	17
*Rhantus manjakatompo* Pederzani & Rocchi, 2009	Yes	43			43
Copelatinae					
*Copelatus ankaratra* Ranarilalatiana & Bergsten, 2019	Yes	233			233
*Copelatus distinguendus* Régimbart, 1903	No	2	32	9	43
*Copelatus lineatipennis* Guignot, 1955	Yes		123	285	408
*Copelatus owas* Régimbart, 1895	Yes	100			100
*Copelatus polystrigus* Sharp, 1882	No	44	14	24	82
*Copelatus ruficapillus* Régimbart, 1895	Yes	5	3	4	12
*Copelatus* sp. n. (*“vazimba”* Ranarilalatiana & Bergsten, in preparation)	Yes	3			3
*Copelatus vigintistriatus* Fairmaire, 1869	No	4	90	1	95
*Madaglymbus* sp.1	Yes		54	92	146
*Madaglymbus* sp.2	Yes			65	65
*Madaglymbus* sp.3	Yes			107	107
*Madaglymbus* sp.4	Yes		41		41
*Madaglymbus* sp.5	Yes		3		3
Cybistrinae					
*Cybister vulneratus* Klug, 1834	No	2	1		3
Dytiscinae					
*Hydaticus bivittatus* Laporte, 1835	No	17			17
*Hydaticus dorsiger* Aubé, 1838	No	41	2	5	48
*Hydaticus exclamationis* Aubé, 1838	No	2			2
*Hydaticus intermedius* Régimbart, 1895	No	10	3	1	14
*Hydaticus limnetes* Guignot, 1955	Yes			27	27
*Hydaticus nigrotaeniatus* Régimbart, 1895	Yes			10	10
*Hydaticus petitii* Aubé, 1838	Yes	1			1
*Hydaticus saecularis* Pederzani, 1982	Yes			1	1
Hydroporinae: Bidessini					
*Bidessus anjozorobe* Bergsten, Ranarilalatiana & Biström, 2020	Yes			2	2
*Bidessus nesioticus* Guignot, 1956	Yes	51			51
*Clypeodytes concivis* Guignot, 1955	Yes	9	20	7	36
*Hydroglyphus flavoguttatus* (Régimbart, 1895)	No	14	7		21
*Hydroglyphus plagiatus* (Kolbe, 1883)	Yes	4			4
*Hydroglyphu*s sp. n.	Yes	9	5		14
*Pachynectes* sp. n.1	Yes		24		24
*Pachynectes* sp. n.2	Yes		1		1
*Pseuduvarus* cf. *vitticollis* (Boheman, 1848)	No*	28	15		43
*Uvarus* cf. *betsimisarakus* (Guignot, 1939)	Yes		7		7
*Uvarus binaghii* Pederzani & Sanfilippo, 1978	Yes			10	10
*Uvarus* cf. *laurentius* Biström 1995	Yes			12	12
*Uvarus* sp. n.1 (“kelycordiformis”)	Yes			21	21
*Uvarus* sp. n.2 (“needle”)	Yes		21		21
*Uvarus* sp. n.3 (“stumpparameres”)	Yes	6			6
*Uvarus* sp. n.4 (“mediotestaceus”)	Yes			1	1
*Uvarus* cf. sp. n.5 (*“dilatatus”* Holmgren et al. in preparation)	Yes		2	6	8
*Uvarus* sp. n.6 (*“cordiformis”* Holmgren et al., in preparation)	Yes			3	3
*Uvarus* sp. n.7 (*“manjakatompo”* Holmgren et al., in preparation)	Yes	406			406
Hydroporinae: Laccornellini					
*Canthyporus reebae* Manuel & Ramahandrison, 2017	Yes	5			5
Hydroporinae: Hydrovatini					
*Hydrovatus contumax* Guignot, 1954	No	1			1
*Hydrovatus dentatus* Bilardo & Rocchi, 1990	No			3	3
*Hydrovatus oblongipennis* Régimbart, 1985	No		7		7
*Hydrovatus otiosus* Guignot, 1945	Yes	1			1
Hydroporinae: Hygrotini					
*Hygrotus laticollis* Fery, 2017	Yes	19			19
*Hygrotus spadiceus* (Sharp, 1882)	Yes	6			6
*Hygrotus travniceki* (Šťastný, 2012)	Yes			12	12
*Hygrotus verticalis* (Sharp, 1882)	Yes	1			1
Hydroporinae: Hyphydrini					
*Hovahydrus perrieri* (Fairmaire, 1898)	Yes		78	7	85
*Hovahydrus* sp. n.1 (“*amfracticoxus*” Englund et al., in preparation)	Yes		1		1
*Hovahydrus* sp. n.2 (“*tempestatibus*” Englund et al., in preparation)	Yes		116		116
*Hovahydrus* sp. n.3 (“brown big”)	Yes		1		1
*Hyphydrus* cf. *cycloides* Régimbart, 1889	No*		2	7	9
*Hyphydrus separandus* Régimbart, 1895	Yes	6		5	11
*Hyphydrus stipes* Sharp, 1882	Yes			1	1
Hydroporinae: Methlini					
*Methles* cf. *cribratellus* (Fairmaire, 1880)	No*		8	2	10
Laccophilinae					
*Africophilus nesiotes* Guignot, 1951	Yes		3		3
*Africophilus pauliani* Legros, 1950	Yes		11		11
*Africophilus* sp. n.1 (“*sauricephalus*” de Jong et al., in preparation)	Yes		34		34
*Africophilus* sp. n.2 (“*ambohitantelyanus*” de Jong et al., in preparation)	Yes		8		8
*Laccophilus alluaudi* Régimbart, 1900	Yes		15		15
*Laccophilus bergsteni* Manuel & Ramahandrison, 2020	Yes	59	5	27	91
*Laccophilus complicatus* Sharp, 1882	Yes	89	13	19	121
*Laccophilus insularum* Biström, Nilsson & Bergsten, 2015	Yes		32	1	33
*Laccophilus lateralis* Sharp, 1882	Yes	18	19	1	36
*Laccophilus luctuosus* Sharp, 1882	Yes			2	2
*Laccophilus olsoufieffi* Guignot, 1937	Yes		18		18
*Laccophilus* sp. *n*. (*“tampoketsa”* Bergsten et al., in preparation)	Yes		3		3
*Philaccolus* sp. n.	Yes		6		6
Gyrinidae					
*Aulonogyrus* (Lophogyrus) *cristatus* Régimbart, 1903	Yes	1			1
*Aulonogyrus* (Pterygyrus) *elegantissimus* Régimbart, 1883	Yes			19	19
*Aulonogyrus* (Paragyrus) *goudoti* Régimbart, 1883	Yes	45	11	114	170
*Dineutus* (Protodineutus) *proximus* Aubé, 1838	Yes	7	33	22	62
*Dineutus* (Protodineutus) *sinuosipennis* Castelnau, 1840	No	4	8	15	27
*Dineutus* (Spinosodineutus) *subspinosus* (Klug, 1834)	No	2		6	8
*Gyrinus madagascariensis* Aubé, 1838	Yes	8	2		10
*Gyrinus* sp. n.	Yes		23		23
*Orectogyrus* (Gonogyrellus) *hastatus* Régimbart, 1892	Yes		1	39	40
*Orectogyrus* (Nesogyrus) *oberthuri* Régimbart, 1884	Yes			6	6
*Orectogyrus* (Meiogyrus) *ornaticollis* (Aubé, 1838)	Yes	8	46	2	56
*Orectogyrus* (Meiogyrus) *sedilloti* Régimbart, 1884	Yes		2	5	7
Haliplidae					
*Peltodytes quadratus* Régimbart, 1895	Yes			1	1
Noteridae					
*Canthydrus concolor* Sharp, 1882	Yes	5		5	10
*Canthydrus* sp.	?		1		1
*Neohydrocoptus* cf. *placidus* (Guignot, 1955)	Yes	8			8
*Neohydrocoptus seriatus* (Sharp, 1882)	No	3		8	11
*Sternocanthus* sp.	?	1	27		28
Total		1436	1003	1024	3463

*Note*: Asterix (*) signifies that the current circumscription of a taxon may be erroneous with an effect on the endemic status.

All three remaining forests housed a remarkable number of undescribed species. A few species, such as *Copelatus ankaratra* from Manjakatompo Ankaratra and *Bidessus anjozorobe* from Anjozorobe‐Angavo were described recently (Bergsten et al., 2020; Ranarilalatiana et al., 2019). Others are in the process of being described and named. New species in the radiations of *Africophilus*, *Hovahydrus*, *Pachynectes*, *Uvarus*, and *Madaglymbus* will be described in larger ongoing revisions of these genera. *Methles* cf. *cribratellus* and *Pseuduvarus* cf. *vitticollis* are complexes with multiple species yet to be disentangled. All three areas had a very high proportion of unique species (i.e., found only within one of the three areas) with Manjakatompo Ankaratra having the highest at 42%, followed by Ambohitantely (40%) and Anjozorobe‐Angavo (38%).

### 3.1 Species assemblages

The single sample from a hygropetric site (collecting event MAD16‐18) was very different and had only four species, of which two were unique for this dataset. This sample was removed from the species assemblages analyses, but the exclusion did not affect the overall results qualitatively. Standing water was sampled in all areas, but had low replicate numbers inside and outside all protected areas. Hence, we did not examine whether the protected area effect is different between running and standing water.

**FIGURE 5:**
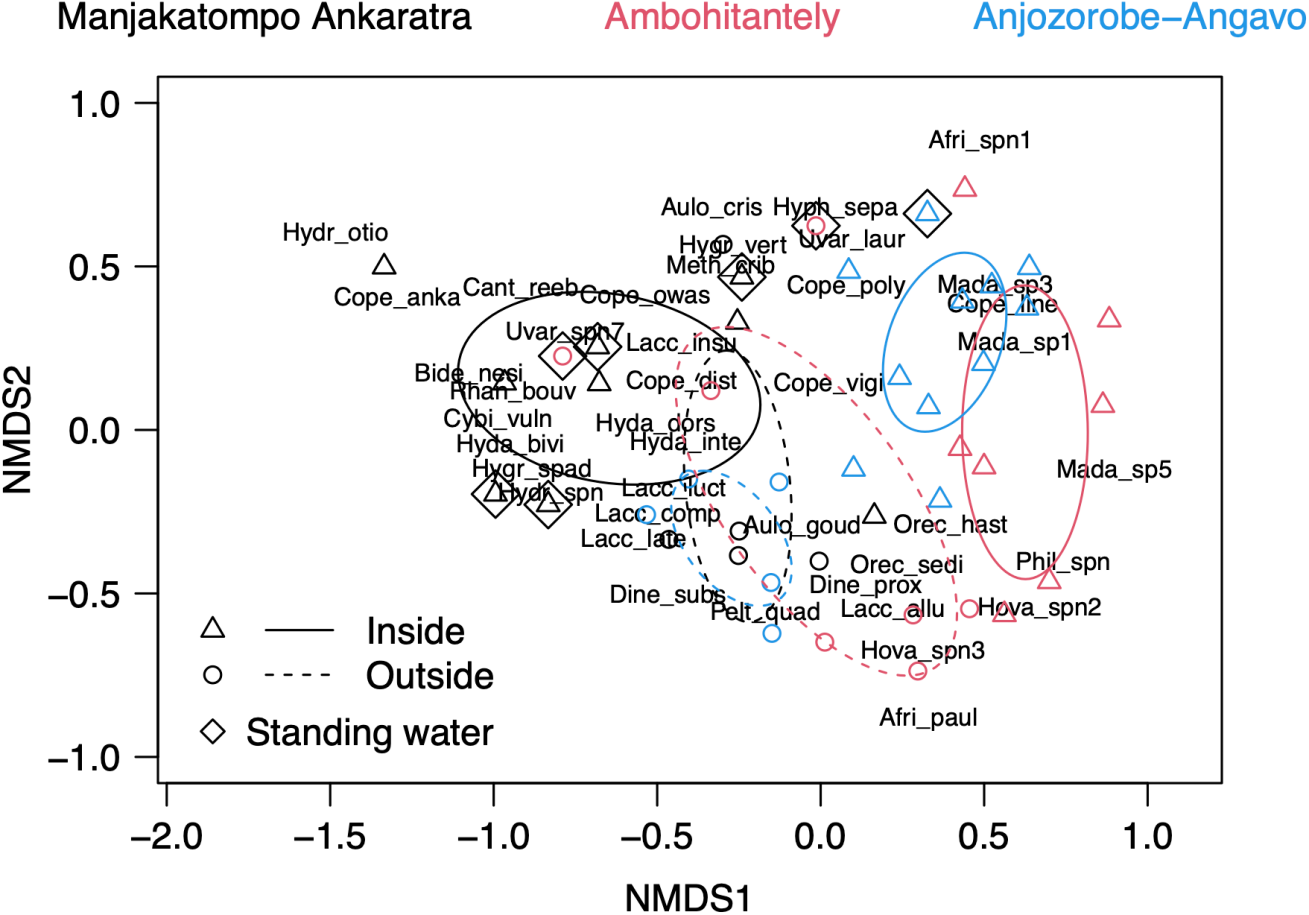
NMDS of beetle species composition in three areas, sampled inside and outside nature reserves. The NMDS analysis shows that outside reserve communities were more similar (located in the center of the NMDS) compared with the inside reserve communities (located on either side of the first axis). Most samples were taken in running waters. Samples from standing waters are indicated in the figure as diamonds enclosing the reserve symbols, and samples from running waters are without diamonds. Ellipses of groups show group centroid and their standard deviation. Species names are eight‐letter abbreviations formed by the first four letters of the genus and four first letters of the species epithet or temporary spn/sp number as given in Table 1. Species names are added as weighted averages of their site abundances, meaning that species are located close to the sites where they had their highest abundance. Only the most abundant species is shown if species names overlap on the ordination.

**FIGURE A1:**
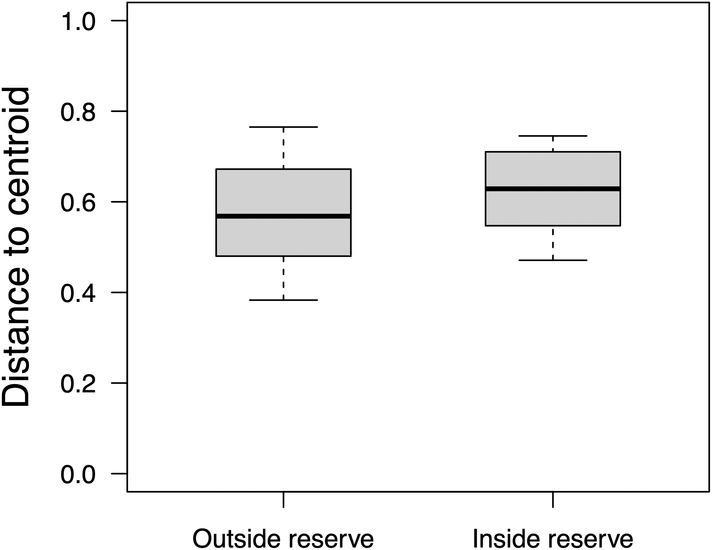
Dispersion boxplot of beetle species dissimilarity matrix grouped into outside and inside nature reserves. Boxplots illustrate the beta‐diversity of the groups as the spread and median distance of the plots to the centroid of each group.

Multivariate analysis using negative binomial models showed that the effect of location (inside vs outside protected area) was different in each area (reserve) (Table 2). The main effect of area and location were overall larger than the interaction effect, supporting the observation that water beetle communities were mainly different among areas, and between inside and outside the reserves independently of area. Similar results were obtained by the PERMANOVA analysis (Table A1). Our NMDS ordination (stress = 0.15) illustrated the separation of the sampled habitats along the two axes (Figure 5). Inside and outside reserve assemblages of beetles differed, but did not dominate any of the axes. Two areas, Ambohitantely and Anjozorobe‐Angavo, showed similar patterns with the distinct grouping of inside and outside the protected area in the ordination. Manjakatompo Ankaratra had a very different species composition inside the protected area compared with the other sites, while the fauna in the peripheral zone was similar among all three areas. The analysis of dissimilarities among sites showed similar beta‐diversity outside and inside reserves (ANOVA: *p* = .12; Figure A1). Manjakatompo Ankaratra had more standing waters sampled but this did not affect the multivariate results.

**TABLE 2:** Multivariate (multi‐species) log‐likelihood ratio tests of generalized linear regression models fitted with negative binomial.

	Res. df	Diff. df	Deviance	Pr(>Dev)
Reserve	40	2	253	<.0001
Location (in/out)	42	1	254	<.0001
Reserve × Location	38	2	122	<.0001

Abbreviations: Diff. df, difference in degrees of freedom; Pr(>Dev), *p*‐value (significance); Res. df, residual degrees of freedom.

**FIGURE 6:**
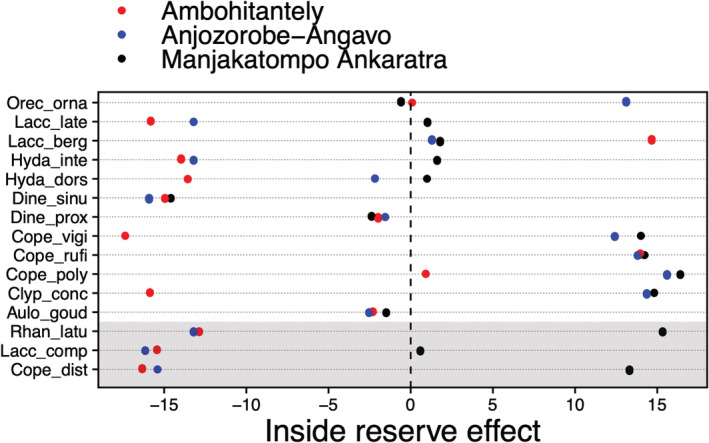
Model estimates of the inside reserve effect compared with outside the reserve on beetle abundance for species recorded in all three areas. Effects are shown for each area as there was a strong multivariate interaction effect between area and location (inside vs outside). Species with a significant Area × Location effect (*p* < .05) are indicated with a shaded area. For species model statistics, see Table A2. Estimates are on the log scale extracted from negative binomial models.

**FIGURE A2:**
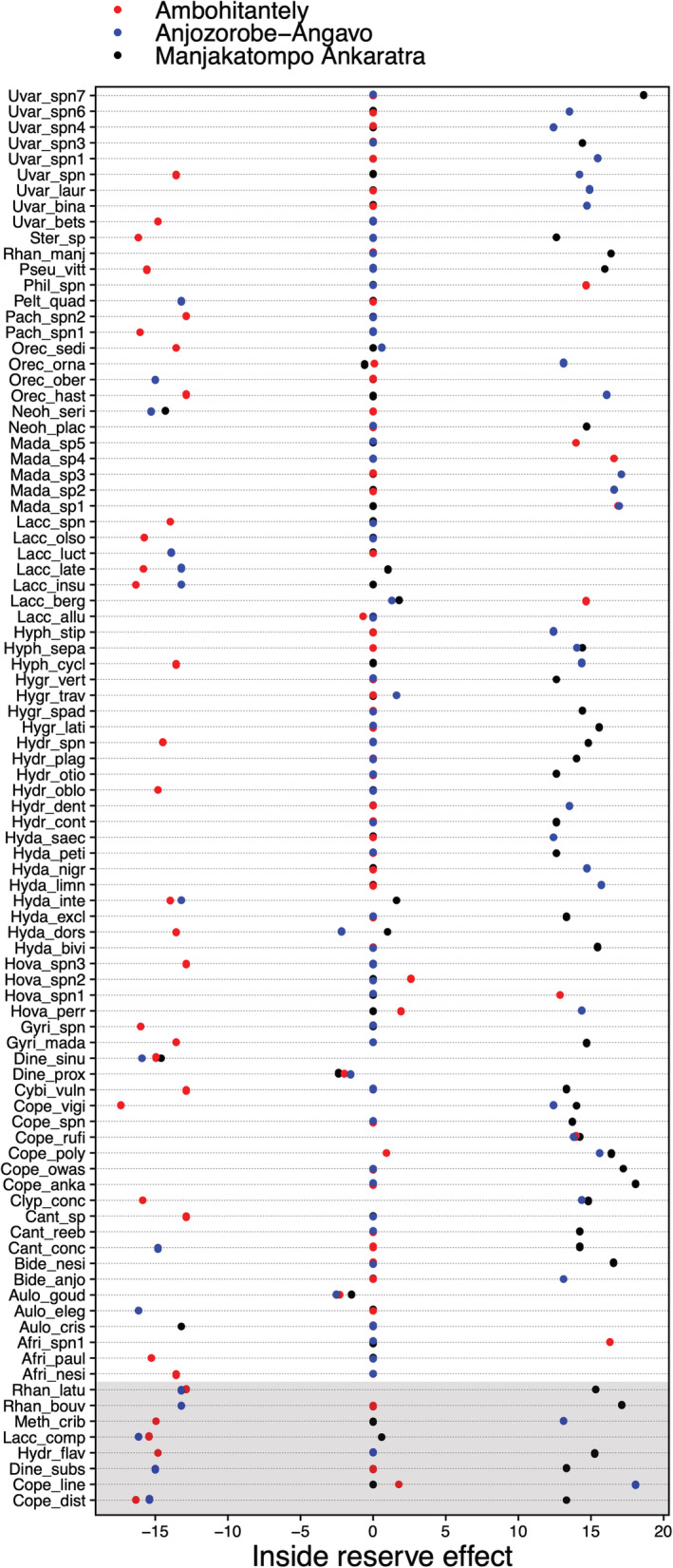
Model estimates of the inside reserve effect compared with outside the reserve on abundance for all species. Effects are shown for each area as there was a strong multivariate interaction effect between area and location (inside vs outside). Species with a significant Area × Location effect (*p* < .05) are indicated with a shaded area. For species model statistics, see Table A2. Estimates are on the log scale extracted from negative binomial models.

Examination of individual species responses detected eight species with an interaction effect (*p* < .05, Figure A2, Table A2). Of those eight species, three species were found in all areas (*Rhantus latus*, *Laccophilus complicatus*, and *Copelatus distinguendus*). In total, 15 species were found in all areas and the abundance of these species was often lower inside the protected areas than outside in the peripheral savannah zone, while no differences or higher abundances inside the reserve was observed at Manjakatompo Ankaratra (Figure 6). Excluding the samples from standing water did not have a major impact on the results but the p‐value for the interaction effect increased slightly for some species, for example, *Laccophilus complicatus* changed from 0.04 to 0.08.

## 4 DISCUSSION

The results of our survey of aquatic beetles show that the Central Highlands of Madagascar is an area of high endemism on Madagascar. We found a surprisingly large number of undescribed highland endemics from the three remaining forests, at least 15, but an exact number is not possible to establish yet as some “cf.” and “sp.” species require more work. New and recently described species that are, as far as known, endemic to the central portions of the highlands are found across a range of diving beetle tribes (Dytiscidae): Bidessini, Hyphydrini, Laccophilini, Copelatini, Laccornellini, Hygrotini, Hydrovatini and one whirligig beetle (Gyrinidae) of the genus *Gyrinus*. Multiple new Bidessini species were found in each of the three remaining forests. Additional taxa like the three endemic *Rhantus* species of Colymbetini are likewise distributed in the Highlands but not restricted to the Central portions (Hjalmarsson et al., 2013). Notably, none of the new species were shared between any two fragments suggesting truly microendemic distributions. The high endemism suggests that the remaining forests of the Central Highlands of Madagascar should be highly prioritized for conservation.

We found that the reserves differed among each other in species assemblages. Thus, from a conservation perspective, any one forest fragment on the Central Highland is not exchangeable or equivalent to any other. We also found that the forest reserves differed in their species assemblages compared with the areas just outside the reserves. However, the community dispersion, which is a measure of beta‐diversity, did not differ between the reserves and areas outside the reserves. In addition, species richness shows no consistent pattern between inside and outside reserves. For Ambohitantely richness was higher outside and for Manjakatompo Ankaratra, it was higher inside protected area boundaries. Such absence of a strong pattern in beta‐diversity and species richness between inside and outside natural forest areas has been found in other insect studies as well (e.g., Navarrete & Halffter, 2008; Niemelä et al., 2007). It can be explained by the fact that dark and cool adapted natural forest species are replaced by warm and open area adapted generalist species (Niemelä et al., 2007; see also below). Nevertheless, other studies have found higher insect diversity inside compared with outside natural forest areas (e.g., Harris & Burns, 2000; Paillet et al., 2010). Despite the absence of a difference in beta‐diversity and species richness in our study, forested habitat areas harbor a more unique assemblage of locally endemic species.

Overall, species assemblages from the open habitats were more similar compared with the variation observed within protected areas (outside vs inside). Assuming that these sites were once forested, this similarity can be seen as a result of two components that drive a process called biotic homogenization (Smart et al., 2006). First, local forest endemics are lost following deforestation, an observation also made for stream insects in eastern Madagascar (Benstead, Douglas, & Pringle, 2003). The endemic genus and island radiation of *Madaglymbus* is one example. Five species of *Madaglymbus* were found in Ambohitantely and Anjozorobe‐Angavo but only at forested sites within the protected areas. *Philaccolus* sp., *Hydaticus nigrotaeniatus* and several of the *Uvarus* species are other examples. Second, these are often replaced by widespread generalist species that either establish or increase in abundance (also see Benstead, Douglas, & Pringle, 2003; Gibon & Elouard, 1996; Irwin et al., 2010). These generalist species are partly nonendemic very widespread African fauna elements such as *Copelatus polystrigus*, *Hydaticus exclamationis*, *H. intermedius*, *H. dorsiger*, *Cybister vulneratus*, *Dineutes subspinosus*, and *Neohydrocoptus seriatus*. From our experience of sampling water beetles throughout Madagascar, these are common species that tolerate some degradation of aquatic habitats. We frequently attract for instance *Neohydrocoptus seriatus* with light traps on land. This indicates they are dispersive and excellent fliers and can rapidly occupy disturbed habitats even in largely forested environments, occasionally even in natural forest canopy gaps but then usually at lower abundances (J. Bergsten, personal observation). The degraded and partly open habitat state within the protected area of Manjakatompo Ankaratra results in a high abundance of, e.g., the generalists *Hydaticus dorsiger* and *H. intermedius*, which in the other two areas are largely found in the open peripheral zone (Tables A6, A7, A8). Generalists may be more tolerant to higher temperatures, siltation, and in situ primary production (Benstead, Douglas, & Pringle, 2003; Benstead & Pringle, 2004).

We did not find general differences in beta‐diversity between inside and outside protected areas (Figure A1). This result is in contrast to our expectations as we would predict a higher level of regionalization, and thereby beta‐diversity in forest‐dwelling taxa. However, there might be several reasons for this unexpected result. In addition to widespread African fauna elements, some Madagascar endemics seem to have adapted to cope with degraded aquatic habitats and are ubiquitous over Madagascar. The gyrinids *Dineutes sinuosipennis* and *Aulonogyrus goudoti* are two examples of this. But there are also candidates of species adapted to natural open or semi‐open habitats. Lentic species occupying wetlands (e.g., some *Hydrovatus*, *Laccophilus*, *Methles*), have generally larger distribution ranges than lotic stream species (Hjalmarsson et al., 2013), are more common in open habitats and may contribute to a more similar savannah fauna. The fact that one site (Manjakatompo Ankaratra) included both alpine and degraded habitats inside the protected area, and the outside sites of Ambohitantely often contained gallery forests and were recently forested (see discussion below) likely contributed to the absence of a beta‐diversity difference. Among the 15 species occurring in all three areas, *Rhantus latus*, *Laccophilus complicatus*, and *Copelatus distinguendus* showed a significant interaction effect and were more abundant inside the reserve in Manjakatompo Ankaratra than outside, but vice versa for the other two areas. This likely reflects the fact that these are quite widespread Central Highland (and beyond) species with a preference for partly open habitats, a type of habitat sampled inside reserve boundaries only in Manjakatompo Ankaratra. There is certainly also a microendemic distribution pattern of non‐forest‐dwelling taxa. The endemic genus *Pachynectes* is an example of a river radiation in Madagascar not found in small forest streams but medium to large size rivers in semi‐open landscapes, and with a significant regionalization of species (Bukontaite et al., 2015). We only found *Pachynectes* outside the protected area of Ambohitantely and not in the other two sites.

Our study did not include chemical water variables like, e.g., pH and nutrients. Obviously, such variables might also affect species abundances and the richness of water beetles. Past studies have shown that the impact of water chemistry on invertebrate diversity differs between study systems, but that landscape variables and pond and stream physical variables always explain a high amount of the aquatic invertebrate diversity variation (Heino et al., 2017; Salvarrey et al., 2014; Thornhill et al., 2017). Thus, based on past studies on aquatic invertebrate diversity, that included landscape and chemical water variables as independent variables, we suggest that our results might also be influenced by chemical water variables among the sampling sites but that landscape variables probably have a major impact as shown in many other studies on aquatic invertebrates. In particular, we note that localities in the peripheral zone of Manjakatompo Ankaratra and Anjozorobe‐Angavo are situated in the vicinity of agricultural areas, or their upstream river catchment conditions involve agricultural areas. This is not the case for localities in the peripheral zone of Ambohitantely, which could partly explain the relatively higher diversity outside the protected area here.

A closer look at the sampled aquatic beetle fauna of Ambohitantely reveals recent forest history in the peripheral grassland zone. The endemic genus *Hovahydrus* consist in our experience of strictly forest‐dwelling clear‐water stream species, with the exception of the montane *H. praetextatus*. This observation is based on 13 years of fieldwork across Madagascar. However, we found *Hovahydrus* outside the protected area of Ambohitantely (localities 25 and 28). Locality 28 turns out to be situated in what was a 30 ha forest fragment at the time Langrand (1995: Fragment “H”) performed his forest fragment bird studies in Ambohitantely. Today a narrow gallery forest remains along the stream. Locality 25 south of the largest remaining forest block was also likely forested not too long ago. The remaining largest forest parcel of today (1160 ha) is approximately 58% of what it was in 1964, and a mere 7% of what it was at the end of the nineteenth century (Langrand, 1995, 2003). Outside the protected area the forested area was reduced by 28% between 1949 and 1992 (Hanssen, 2002). All our sites at Ambohitantely were likely forested and part of the estimated 20 × 8 km large forest block in 1897 (Langrand, 1995, 2003). This, together with some remaining gallery forests along streams, could explain a more species‐rich fauna outside the protected area in Ambohitantely. The remaining gallery forests seem sufficient for at least short‐term survival and occupancy even of forest‐dwelling taxa, at the same time as open habitat taxa move in. However, the long‐term survival here of *Hovahydrus* is uncertain as these stream‐gallery forests outside protected areas are continually shrinking from yearly fires.

The vast majority of biodiversity surveys in Madagascar have focused on largely undisturbed forests in the eastern evergreen moist forest realm or the western dry deciduous forests. The Central Highlands forest pockets, surrounded by a heavily fire‐altered grassland landscape, have been assumed to be locally poor in diversity (Andreone et al., 2007). A recent prominent theory to explain the microendemism pattern in Madagascar predicted higher elevations to contain proportionally lower levels of microendemism (Wilmé et al., 2006). But, especially in the herpetological literature (notably Brown et al., 2014; Raxworthy & Nussbaum, 1996), an endemic montane fauna was acknowledged, with a focus on the three highest peaks of the Highlands, Tsaratanana, Ankaratra, and Andringitra. Treeless secondary montane heathland was rejected as recent anthropogenic habitats, albeit fire‐affected, as they supported a number of montane endemic amphibians and reptiles. Our findings on water beetles add to the knowledge that the montane heathlands of Madagascar are areas of high endemism. We found that Manjakatompo Ankaratra had the most unique fauna of the three forest fragments, while the two northern forests Ambohitantely and Anjozorobe‐Angavo had a more similar fauna. This makes sense from a geographical viewpoint: Ambohitantely and Anjororobe‐Angavo are situated approximately 70 km apart whereas Manjakatompo Ankaratra lies further south at a geographical distance of almost double. Perhaps more importantly, Manjakatompo Ankaratra has a much larger altitude span that can support a wider range of taxa as water beetles are niche differentiated along the altitude gradient. In Manjakatompo Ankaratra we sampled montane habitats above 2000 m, which does not exist in Ambohitantely or Anjozorobe‐Angavo. *Copelatus ankaratra*, *Canthyporus reebae*, and *Bidessus nesioticus* are examples of high‐altitude montane species we have only found above 2000 m, the first two endemic to Manjakatompo Ankaratra. The higher elevation zone at Ankaratra with multiple vertebrate and invertebrate endemics is thus an important conservation priority for montane habitats and species (Andreone et al., 2014; Goodman et al., 1996; Hjalmarsson et al., 2013; Ranarilalatiana et al., 2019; Vences et al., 2002).

Previous studies on Vertebrates also found Ambohitantely to have a comparable species composition to Anjozorobe‐Angavo. The communities were either similar (Lipotyphlan mammals; Goodman & Rakotondravony, 2000) or had a depauperate but nested fauna compared with the larger neighboring forest (birds; Langrand & Wilmé, 1997). There are no bird species locally endemic to any of the three sites, but one mammal species (a Nesomyid rodent, *Voalavo antsahabensis*) is solely known from Anjozorobe‐Angavo (Goodman et al., 2018). Among amphibians, there are three species endemic to Ambohitantely (*Boophis andrangoloaka*, *Anilany helenae*, and *Anodonthyla vallani*), and our study adds a surprisingly large number of new species only known from here. Given the small remnant nature of the area, this is clearly a hotspot of irreplaceable midaltitude (1500–1700 m) Central Highlands fauna.

All three areas have experienced forest cover loss during the last two decades (1996–2016; Goodman et al., 2018). Ambohitantely has been least affected in this time window (but see Langrand, 1995, 2003 for older exploitation), while for Anjozorobe‐Angavo and Manjakatompo Ankaratra, 20%–30% disappeared (Goodman et al., 2018). With the loss of a large percentage of the natural forest extent, it is possible that the endemic fauna of water beetles are at extinction risk since small forest reserves might have an extinction debt (Tilman et al., 1994). In addition, very little is known about the required habitat size for the long‐term survival of these taxa. Therefore, conservation efforts should be directed towards the prevention of further degradation and habitat loss, as well as forest regeneration near margins (Pareliussen et al., 2006). In addition, localized endemics are at high risk of extinction from irregular events, such as the introduction of fish or if extreme drought would completely dry out normally perennial streams.

In conclusion, the loss of remaining forests of the Madagascar Central Highlands would depauperate the aquatic insect fauna on the island. The highlands are rich in local midaltitude endemics and vastly undersampled for insects. We found a remarkable number of undescribed species across a range of water beetle tribes and in all three areas. None of these were shared between any two forest fragments. In the short term, some forest‐dwelling aquatic taxa can survive in gallery forest‐lined water courses, but in the longer term, deforested aquatic habitats are characterized by a more similar fauna across geographical space. Ambohitantely is the last chance of retaining a >1000 ha block of 1500–1700 m midaltitude Central Highland forests with unique biodiversity. For Manjakatompo Ankaratra, the secondary forest of mixed native/exotic trees has probably prevented the local extinction of microendemic aquatic taxa and this site also harbors unique montane fauna elements in the alpine zone >2000 m. Anjozorobe‐Angavo is the largest forest block of the three forests investigated, but also the one experiencing the highest pressure and has lost 33% since 1996. Also in this forest we found local endemics, which emphasize the high priority of further biological inventories, and predict that more endemics would be revealed. This supports the view that we are still just scratching the surface of invertebrate richness in Madagascar.

## AUTHOR CONTRIBUTIONS

**Tolotra Ranarilalatiana:** Conceptualization (equal); data curation (equal); investigation (equal); methodology (equal); writing – original draft (equal); writing – review and editing (equal). **Herisolo Andrianiaina Razafindraleva:** Writing – review and editing (equal). **Gustaf Granath:** Formal analysis (equal); methodology (equal); software (equal); visualization (equal); writing – original draft (equal); writing – review and editing (equal). **Rasa Bukontaite Malm:** Visualization (equal); writing – review and editing (equal). **Jean Claude Rakotonirina:** Writing – review and editing (equal). **Victor Razafindranaivo:** Writing – review and editing (equal). **Lala Harivelo Raveloson Ravaomanarivo:** Supervision (equal); writing – review and editing (equal). **Frank Johansson:** Conceptualization (equal); data curation (equal); methodology (equal); writing – original draft (equal); writing – review and editing (equal). **Johannes Bergsten:** Conceptualization (equal); funding acquisition (equal); methodology (equal); project administration (equal); supervision (equal); validation (equal); writing – original draft (equal); writing – review and editing (equal).

## FUNDING INFORMATION

This work was supported by the Swedish Research Council (Grant numbers 2009‐3744 and 2013‐517) and the Royal Swedish Academy of Sciences to JB.

## CONFLICT OF INTEREST

The authors have no relevant financial or nonfinancial interests to disclose.

## Data Availability

The dataset generated and analyzed during the current study is available from Zenodo (https://doi.org/10.5281/zenodo.7118949) together with explanatory metadata. Tables A3, A4, A5, A6, A7, A8 include locality details, species occupancy, and summed abundances.

## APPENDIX 1

**TABLE A1:** PERMANOVA results on species abundances based on 1999 permutations.

	df	SS	MS	*F*	*R* ^2^	Pr(>*F*)
Reserve	2	2.25	1.13	3.3	.12	<.0001
Location (in/out)	1	1.70	1.70	5.0	.09	<.0001
Reserve × Location	2	1.23	0.62	1.8	.07	.011
Residuals	38	12.94	0.34		.71	
Total	43	18.13			1	

Abbreviations: df, Degrees of freedom; *F*, *F*‐value (*F* statistic); MS, mean sum of squares; Pr(<*F*), *p*‐value (significance); *R* ^2^, *R*‐squared; SS, sum of squares.

**TABLE A2:** Univariate log‐likelihood ratio tests of generalized linear regression models fitted with negative binomial for all species.

Species	Area	Location	Area:Location	No. of samples with species recorded
Rhan_bouv	0.003	0.619	0.007	7
Rhan_latu	0.029	0.710	0.002	7
Rhan_manj	0.083	0.362	0.448	2
Cope_anka	0.062	0.246	0.306	2
Cope_poly	0.699	0.044	0.145	11
Cope_dist	0.215	0.017	0.005	8
Cope_line	0.001	0.004	0.024	16
Cope_owas	0.003	0.076	0.362	4
Cope_rufi	0.939	0.071	0.952	4
Cope_spn	0.049	0.265	0.691	2
Cope_vigi	0.382	0.919	0.101	4
Mada_sp1	0.043	0.002	0.479	9
Mada_sp2	0.001	0.001	0.444	9
Mada_sp3	0.004	0.056	0.737	5
Mada_sp4	0.002	0.002	0.370	5
Mada_sp5	0.075	0.073	0.207	2
Hyda_bivi	0.003	0.027	0.608	5
Hyda_dors	0.006	0.450	0.096	13
Hyda_excl	0.048	0.233	0.615	2
Hyda_inte	0.356	0.453	0.141	6
Hyda_limn	0.001	0.021	0.373	6
Hyda_nigr	0.011	0.098	0.614	4
Hyda_peti	0.335	0.293	0.390	1
Hyda_saec	0.414	0.451	0.650	1
Cybi_vuln	0.516	0.916	0.135	2
Afri_nesi	0.185	0.150	0.212	1
Afri_paul	0.158	0.356	0.302	1
Afri_spn1	0.283	0.119	0.308	1
Lacc_berg	0.132	0.026	0.575	15
Lacc_allu	0.013	0.690	0.750	3
Lacc_spn	0.246	0.298	0.516	1
Lacc_comp	0.043	0.012	0.016	13
Lacc_late	0.395	0.243	0.178	6
Lacc_luct	0.377	0.075	0.343	1
Lacc_insu	0.053	0.017	0.606	4
Lacc_olso	0.220	0.314	0.354	1
Phil_spn	0.081	0.075	0.291	2
Bide_nesi	0.232	0.487	0.571	1
Bide_anjo	0.386	0.241	0.304	1
Clyp_conc	0.710	0.683	0.131	4
Hydr_flav	0.067	0.780	0.017	6
Hydr_plag	0.021	0.075	0.450	3
Hydr_spn	0.593	0.811	0.173	2
Pach_spn1	0.063	0.065	0.513	2
Pach_spn2	0.155	0.408	0.531	1
Pseu_vitt	0.239	0.661	0.074	4
Uvar_bina	0.123	0.282	0.315	2
Uvar_bets	0.326	0.376	0.416	1
Uvar_spn	0.681	0.847	0.185	2
Uvar_laur	0.389	0.485	0.548	1
Uvar_spn6	0.034	0.155	0.432	3
Uvar_spn7	0.125	0.374	0.177	2
Uvar_spn1	0.489	0.409	0.359	1
Uvar_spn3	0.154	0.235	0.268	1
Uvar_spn4	0.414	0.378	0.201	1
Cant_reeb	0.014	0.079	0.262	3
Hydr_cont	0.246	0.473	0.467	1
Hydr_dent	0.405	0.369	0.437	1
Hydr_oblo	0.076	0.166	0.284	2
Hydr_otio	0.249	0.317	0.644	1
Hygr_vert	0.317	0.512	0.632	1
Hygr_lati	0.016	0.143	0.401	3
Hygr_spad	0.082	0.283	0.295	2
Hygr_trav	0.158	0.700	0.595	2
Hova_spn1	0.200	0.125	0.626	1
Hova_spn2	0.002	0.237	0.545	5
Hova_perr	0.154	0.367	0.387	4
Hova_spn3	0.155	0.487	0.534	1
Hyph_cycl	0.667	0.864	0.232	2
Hyph_sepa	0.631	0.283	0.841	2
Hyph_stip	0.414	0.319	0.215	1
Meth_crib	0.147	0.464	0.029	4
Aulo_cris	0.327	0.108	0.513	1
Aulo_eleg	0.036	0.004	0.280	3
Aulo_goud	0.065	0.002	0.816	19
Dine_prox	0.230	0.002	0.879	17
Dine_subs	0.298	0.372	0.042	3
Dine_sinu	0.696	0.001	0.279	8
Gyri_mada	0.475	0.732	0.135	2
Gyri_spn	0.145	0.255	0.078	1
Orec_hast	0.379	0.892	0.179	2
Orec_sedi	0.349	0.690	0.220	3
Orec_ober	0.127	0.021	0.452	2
Orec_orna	0.013	0.910	0.507	11
Pelt_quad	0.485	0.069	0.590	1
Cant_conc	0.395	0.593	0.052	3
Cant_sp	0.170	0.490	0.644	1
Ster_sp	0.170	0.543	0.144	3
Neoh_plac	0.259	0.494	0.422	1
Neoh_seri	0.675	0.029	0.752	2

*Note*: Numbers are *p*‐values, Pr (>Deviance), testing the effect of area, location within the area (inside versus outside reserve), and their interaction. Number of samples where a species was recorded is indicated in the last column.

**TABLE A3:** Manjakatompo Ankaratra sampling localities and habitats.

Field_ID	Lat (N)	Lon (E)	Elev (m)	Habitat and locality	Status
MAD16‐01	−19.37588	47.3518	1599	Andalantsoavaly stream	Outside
MAD16‐02	−19.34409	47.32322	1796	Side pools in Ankaratra stream, up the cascade	Reserve
MAD16‐03	−19.34292	47.33893	1651	Lake with grass at margins, “Lac froid”	Reserve
MAD16‐04	−19.35443	47.32719	1684	Andoharanovelona stream	Outside
MAD16‐05	−19.34917	47.27736	2044	Stream with grass near the source, Tavolotara forest	Reserve
MAD16‐06	−19.36397	47.29907	1759	Large pond near Analamitana stream	Reserve
MAD16‐07	−19.36831	47.29921	1725	Down the stream Analamitana	Outside
MAD16‐08	−19.34505	47.30384	2062	Large pond with grass in stream source, Anosiarivo forest	Reserve
MAD16‐09	−19.35898	47.29994	1315	Swamp with grass, Tandraholatra	Reserve
MAD16‐10	−19.33753	47.2453	2466	Stream with side pools and grass at edge, Ankafotra	Reserve
MAD16‐11	−19.35163	47.24278	2597	Small stream with grass near the source, Tsiafajavona	Reserve
MAD16‐12	−19.37001	47.27005	2000	Open marsh with grass, Analamisaraka	Reserve
MAD16‐13	−19.38426	47.29574	1735	Ankitsikitsika stream	Outside
MAD16‐14	−19.37657	47.34349	1600	Ankeniheny stream	Outside

*Note*: Status indicates if the locality is situated inside or outside of the reserve boundaries.

**TABLE A4:** Ambohitantely sampling localities and habitats.

Field_ID	Lat (N)	Lon (E)	Elev (m)	Habitat and locality	Status
MAD16‐15	−18.22367	47.27758	1485	Pools in forest stream, east of Vatobe	Reserve
MAD16‐16	−18.22053	47.27651	1524	Hygropetric, rockpool in forest stream	Reserve
MAD16‐17	−18.2234	47.28975	1418	Forest stream up the cascade	Reserve
MAD16‐18	−18.1851	47.30462	1495	Hygropetric, rockpools in forest stream	Reserve
MAD16‐19	−18.18411	47.30251	1533	Forest stream	Reserve
MAD16‐20	−18.18074	47.29021	1482	Forest stream down the cascade	Reserve
MAD16‐21	−18.16204	47.2888	1438	Forest stream	Reserve
MAD16‐22	−18.16321	47.28335	1553	Side pools in large forest stream	Reserve
MAD16‐23	−18.09385	47.27145	1346	Stream with bedrock in savannah down the Manjato forest	Outside
MAD16‐24	−18.13132	47.23976	1551	Lake with grass at margins in the road to Manjato forest	Outside
MAD16‐25	−18.25389	47.29303	1178	Ambarifafy stream with rock, bedrock, cascade	Outside
MAD16‐26	−18.22102	47.25087	1547	Large stream by the road to military camp, bog at edge	Outside
MAD16‐27	−18.08130	47.32544	1270	Ambatofotsiloha stream	Outside
MAD16‐28	−18.16296	47.25992	1462	Marofitatra stream	Outside
MAD16‐29	−18.16717	47.26090	1532	Bog in Southwest of Ambohitantely reserve	Outside

*Note*: Status indicates if the locality is situated inside or outside of the reserve boundaries.

**TABLE A5:** Anjozorobe‐Angavo sampling localities and habitats.

Field_ID	Lat (N)	Lon (E)	Elev (m)	Habitat and locality	Status
MAD16‐30	−18.47317	47.94142	1259	Sahavilana Stream	Outside
MAD16‐31	−18.45559	47.95046	1308	Forest stream with sidepools, Ankodondona	Reserve
MAD16‐32	−18.45036	47.94982	1306	Forest stream with sidepools, Antavinamboa	Reserve
MAD16‐33	−18.45113	47.94677	1298	Forest stream with sidepools, Ambatonisana	Reserve
MAD16‐34	−18.4515	47.96317	1424	Forest pond with dead leaves, Sahalemaka	Reserve
MAD16‐35	−18.44919	47.95928	1367	Forest stream with sidepools, Sahalemaka	Reserve
MAD16‐36	−18.45792	47.93438	1377	Ambatovikinina stream	Outside
MAD16‐37	−18.42555	47.95121	1331	Forest stream with sidepools, Ankorona	Reserve
MAD16‐38	−18.42407	47.9559	1356	Forest stream with sidepools, Andrianarivo	Reserve
MAD16‐39	−18.42192	47.93801	1308	Forest stream with sidepools, South of Angavo	Reserve
MAD16‐40	−18.41266	47.9438	1341	Marsh and small forest stream, Circuit bambou	Reserve
MAD16‐41	−18.42824	47.92887	1258	Ambohimiadana stream	Outside
MAD16‐42	−18.47284	47.95938	1401	Forest stream with cascade, rockpools, Ambohimanga	Reserve
MAD16‐43	−18.4671	47.93807	1271	Stream with side pools, Antanambe	Outside
MAD16‐44	−18.4676	47.92535	1271	Small stream, bedrock, and grass at edge, Mangarivotra	Outside
MAD16‐45	‐18.40895	47.9412	1314	Forest stream with sidepools, North of Amoromena	Reserve

*Note*: Status indicates if the locality is situated inside or outside of the reserve boundaries.

**TABLE A6:** Species of aquatic Adephaga collected inside and outside of Manjakatompo Ankaratra reserve in 2016

Taxon	Localities inside reserve	Nb	Localities outside reserve	Nb
Dytiscidae				
*Rhantus bouvieri* Régimbart, 1900	03, 08, 09, 10, 11, 12	90		
*Rhantus latus* (Fairmaire, 1869)	05, 08, 09, 10, 12	15		
*Rhantus manjakatompo* Pederzani & Rocchi, 2009	05, 06	43		
*Copelatus ankaratra* Ranarilalatiana & Bergsten, 2019	08, 11	233		
*Copelatus polystrigus* Sharp, 1882	05, 06, 09, 10	44		
*Copelatus distinguendus* Régimbart, 1903	03, 10	2		
*Copelatus owas* Régimbart, 1895	06, 08, 09, 10	100		
*Copelatus ruficapillus* Régimbart, 1895	05	5		
*Copelatus* sp. *n*. (*“vazimba”* Ranarilalatiana & Bergsten, in preparation)	05, 10	3		
*Copelatus vigintistriatus* Fairmaire, 1869	02	4		
*Hydaticus bivittatus* Laporte, 1835	03, 08, 09, 10, 12	17		
*Hydaticus dorsiger* Aubé, 1838	06, 08, 09, 10, 12	34	01, 07, 13	7
*Hydaticus exclamationis* Aubé, 1838	03, 09	2		
*Hydaticus intermedius* Régimbart, 1895	06, 09	9	14	1
*Hydaticus petitii* Aubé, 1838	09	1		
*Cybister vulneratus* Klug, 1834	03	2		
*Laccophilus bergsteni* Manuel & Ramahandrison, 2020	02, 05, 06	54	01	5
*Laccophilus complicatus* Sharp, 1882	03, 08, 09, 10, 12	68	04, 07, 13, 14	21
*Laccophilus lateralis* Sharp, 1882	03, 12	17	07, 14	3
*Bidessus nesioticus* Guignot, 1956	08	51		
*Clypeodytes concivis* Guignot, 1955	03	9		
*Hydroglyphus flavoguttatus* (Régimbart, 1895)	05, 08, 09, 11	14		
*Hydroglyphus plagiatus* (Kolbe, 1883)	08, 09, 10	4		
*Hydroglyphus* sp. *n*.	12	9		
*Pseuduvarus* cf. *vitticollis* (Boheman, 1848)	09, 10, 11	28		
*Uvarus* sp. *n.3* (“stumpparameres”)	08	6		
*Uvarus* sp. *n.7* (*“manjakatompo”* Holmgren et al., in preparation)	08, 09	406		
*Canthyporus reebae* Manuel & Ramahandrison, 2017	05, 10, 11	5		
*Hydrovatus contumax* Guignot, 1954	03	1		
*Hydrovatus otiosus* Guignot, 1945	11	1		
*Hygrotus verticalis* (Sharp, 1882)	06	1		
*Hygrotus laticollis* Fery, 2017	05, 10, 11	19		
*Hygrotus spadiceus* (Sharp, 1882)	09, 12	6		
*Hyphydrus separandus* Régimbart, 1895	06	6		
Gyrinidae				
*Aulonogyrus* (Lophogyrus) *cristatus* Régimbart, 1903			01	1
*Aulonogyrus* (Paragyrus) *goudoti* Régimbart, 1883	02, 05	13	04, 07, 13, 14	32
*Dineutus* (Protodineutus) *proximus* Aubé, 1838	02	1	04, 13, 14	6
*Dineutus* (Spinosodineutus) *subspinosus* (Klug, 1834)	03	2		
*Dineutus* (Protodineutus) *sinuosipennis* Castelnau, 1840			13, 14	4
*Gyrinus madagascariensis* Aubé, 1838	03	8		
*Orectogyrus* (Meiogyrus) *ornaticollis* (Aubé, 1838)	02	4	04	4
Noteridae				
*Canthydrus concolor* Sharp, 1882	03, 10	5		
*Sternocanthus* sp.	03	1		
*Neohydrocoptus* cf. *placidus* (Guignot, 1955)	03	8		
*Neohydrocoptus seriatus* (Sharp, 1882)			13	3
Total abundance		1320		116
Species richness		42		12

**TABLE A7:** Species of aquatic Adephaga collected inside and outside of Ambohitantely reserve in 2016

Taxon	Reserve	Nb	Out reserve	Nb
Dytiscidae				
*Rhantus latus* (Fairmaire, 1869)			24	1
*Copelatus polystrigus* Sharp, 1882	15, 16	10	26, 29	4
*Copelatus distinguendus* Régimbart, 1903			24, 26, 29	32
*Copelatus lineatipennis* Guignot, 1955	15, 16, 19, 21, 22	105	26, 29	18
*Copelatus ruficapillus* Régimbart, 1895	15, 16	3		
*Copelatus vigintistriatus* Fairmaire, 1869			26, 29	90
*Madaglymbus* sp.1	20, 21	54		
*Madaglymbus* sp.4	15, 16, 19, 21, 22	41		
*Madaglymbus* sp.5	19, 20	3		
*Hydaticus dorsiger* Aubé, 1838			24	2
*Hydaticus intermedius* Régimbart, 1895			24, 26	3
*Cybister vulneratus* Klug, 1834			24	1
*Africophilus nesiotes* Guignot, 1951	18	1	23	2
*Africophilus pauliani* Legros, 1950			23	11
*Africophilu*s sp. *n.1* (“*sauricephalus*” de Jong et al., in preparation)	16, 18	34		
*Africophilus* sp. *n.2* (“*ambohitantelyanus*” de Jong et al., in preparation)	18	8		
*Laccophilus alluaudi* Régimbart, 1900	22	5	25, 27	10
*Laccophilus bergsteni* Manuel & Ramahandrison, 2020	19, 22	5		
*Laccophilus* sp. *n*. (*“tampoketsa”* Bergsten et al., in preparation)			26	3
*Laccophilus complicatus* Sharp, 1882			24, 26	13
*Laccophilus lateralis* Sharp, 1882			26	19
*Laccophilus insularum* Biström, Nilsson & Bergsten, 2015			24, 26, 29	32
*Laccophilus olsoufieffi* Guignot, 1937			26	18
*Philaccolus* sp. *n*.	17, 19	6		
*Clypeodytes concivis* Guignot, 1955			26, 29	20
*Hydroglyphus flavoguttatus* (Régimbart, 1895)			24, 26	7
*Hydroglyphus* sp. *n*.			26	5
*Pachynectes* sp. *n*.1			25, 26	24
*Pachynectes* sp. *n*.2			25	1
*Pseuduvarus* cf. *vitticollis* (Boheman, 1848)			24	15
*Uvarus* cf. *betsimisarakus* (Guignot, 1939)			26	7
*Uvarus* sp. *n*.2 (“needle”)	18	21		
*Uvarus* cf. sp. *n*.5 (*“dilatatus”* Holmgren et al., in preparation)			24	2
*Hydrovatus oblongipennis* Régimbart, 1985			26, 29	7
*Hovahydrus* sp. *n*.1 (“*amfracticoxus*” Englund et al., in preparation)	20	1		
*Hovahydrus* sp. *n*.2 (“*tempestatibus*” Englund et al., in preparation)	17, 19, 20	108	25, 28	8
*Hovahydrus perrieri* (Fairmaire, 1898)	20	68	25, 28	10
*Hovahydrus* sp. *n*.3 (“brown big”)			25	1
*Hyphydrus* cf. *cycloides* Régimbart, 1889			26	2
*Methles* cf. *cribratellus* (Fairmaire, 1880)			24, 26, 29	8
Gyrinidae				
*Aulonogyrus* (Paragyrus) *goudoti* Régimbart, 1883	22	1	23, 25, 27	10
*Dineutus* (Protodineutus) *proximus* Aubé, 1838	15, 17, 20	4	25, 26, 27, 28	29
*Dineutus* (Protodineutus) *sinuosipennis* Castelnau, 1840			23, 25, 26	8
*Gyrinus madagascariensis* Aubé, 1838			26	2
*Gyrinus* sp. *n*.			26	23
*Orectogyrus* (Gonogyrellus) *hastatus* Régimbart, 1892			25	1
*Orectogyrus* (Meiogyrus) *sedilloti* Régimbart, 1884			25	2
*Orectogyrus* (Meiogyrus) *ornaticollis* (Aubé, 1838)	15, 17, 20, 22	24	23, 25, 28	22
Noteridae				
*Canthydrus* sp.			26	1
*Sternocanthus* sp.			24, 26	27
Total abundance		502		501
Species richness		18		39

**TABLE A8:** Species of aquatic Adephaga collected inside and outside Anjozorobe‐Angavo reserve in 2016

Taxon	Reserve	Nb	Out reserve	Nb
Dytiscidae				
*Rhantus bouvieri* Régimbart, 1900			43	1
*Rhantus latus* (Fairmaire, 1869)			44	1
*Copelatus polystrigus* Sharp, 1882	34, 40, 42	24		
*Copelatus distinguendus* Régimbart, 1903			36, 43, 44	9
*Copelatus lineatipennis* Guignot, 1955	31, 33, 34, 35, 37, 38, 39, 40, 45	285		
*Copelatus ruficapillus* Régimbart, 1895	34	4		
*Copelatus vigintistriatus* Fairmaire, 1869	34	1		
*Madaglymbus* sp.*1*	32, 33, 37, 38, 39, 40, 45	92		
*Madaglymbus* sp.*2*	31, 33, 34, 35, 37, 38, 40, 42, 45	65		
*Madaglymbus* sp.*3*	35, 37, 38, 40, 45	107		
*Hydaticus dorsiger* Aubé, 1838	40	1	30, 43, 44	4
*Hydaticus intermedius* Régimbart, 1895			36	1
*Hydaticus limnetes* Guignot, 1955	33, 34, 35, 37, 39, 40	27		
*Hydaticus nigrotaeniatus* Régimbart, 1895	33, 34, 39, 45	10		
*Hydaticus saecularis* Pederzani, 1982	40	1		
*Laccophilus bergsteni* Manuel & Ramahandrison, 2020	32, 33, 35, 37, 38, 39, 40, 45	24	36	3
*Laccophilus complicatus* Sharp, 1882			36, 43	19
*Laccophilus lateralis* Sharp, 1882			30	1
*Laccophilus luctuosus* Sharp, 1882			44	2
*Laccophilus insularum* Biström, Nilsson & Bergsten, 2015			44	1
*Bidessus anjozorobe* Bergsten, *Ranarilalatiana & Biström* 2020	40	2		
*Clypeodytes concivis* Guignot, 1955	40	7		
*Uvarus binaghii* Pederzani & Sanfilippo, 1978	34, 39	10		
*Uvarus* cf. *laurentius* Biström 1995	40	12		
*Uvarus* sp. *n.1* (“kelycordiformis”)	38	21		
*Uvarus* sp. *n.4* (“mediotestaceus”)	40	1		
*Uvarus* cf. sp. *n*.5 (*“dilatatus”* Holmgren et al., in preparation)	40	6		
*Uvarus* sp. *n.6* (*“cordiformis”* Holmgren et al., in preparation)	33, 35, 37	3		
*Hydrovatus dentatus* Bilardo & Rocchi, 1990	40	3		
*Hygrotus travniceki* (Šťastný, 2012)	40	11	36	1
*Hovahydrus perrieri* (Fairmaire, 1898)	37	7		
*Hyphydrus* cf. *cycloides* Régimbart, 1889	40	7		
*Hyphydrus separandus* Régimbart, 1895	34	5		
*Hyphydrus stipes* Sharp, 1882	40	1		
*Methles* cf. *cribratellus* (Fairmaire, 1880)	40	2		
Gyrinidae				
*Aulonogyrus* (Pterygyrus) *elegantissimus* Régimbart, 1883			30, 36, 41	19
*Aulonogyrus* (Paragyrus) *goudoti* Régimbart, 1883	31, 32, 37, 39	17	30, 36, 41, 43, 44	97
*Dineutus* (Protodineutus) *proximus* Aubé, 1838	32, 39, 42	7	30, 36, 41	15
*Dineutus* (Spinosodineutus) *subspinosus* (Klug, 1834)			41, 43	6
*Dineutus* (Protodineutus) *sinuosipennis* Castelnau, 1840			36, 41, 44	15
*Orectogyrus* (Gonogyrellus) *hastatus* Régimbart, 1892	42	39		
*Orectogyrus* (Meiogyrus) *sedilloti* Régimbart, 1884	42	4	30	1
*Orectogyrus* (Nesogyrus) *oberthuri* Régimbart, 1884			30, 36	6
*Orectogyrus* (Meiogyrus) *ornaticollis* (Aubé, 1838)	37, 42	2		
Haliplidae				
*Peltodytes quadratus* Régimbart, 1895			30	1
Noteridae				
*Canthydrus concolor* Sharp, 1882			43	5
*Neohydrocoptus seriatus* (Sharp, 1882)			43	8
Total abundance		808		216
Species richness		32		21
